# Improved detectability of acute and subacute brainstem infarctions by combining standard axial and thin-sliced sagittal DWI

**DOI:** 10.1371/journal.pone.0200092

**Published:** 2018-07-03

**Authors:** Michael H. Schönfeld, Robert M. Ritzel, Andre Kemmling, Marielle Ernst, Jens Fiehler, Susanne Gellißen

**Affiliations:** 1 Department of Diagnostic and Interventional Neuroradiology, University Medical Center Hamburg-Eppendorf, Hamburg, Germany; 2 Department of Radiology, German Armed Forces Hospital Hamburg, Hamburg, Germany; 3 Department of Neuroradiology, University Hospital of Luebeck, Luebeck, Germany; Universitatsklinikum Freiburg, GERMANY

## Abstract

**Background and purpose:**

Most false negative findings in DWI of ischemic stroke are in patients with minor deficits clinically localized to the brainstem. Our goal was to evaluate the benefit of a thin-sliced sagittal DWI in addition to conventional axial DWI at 1.5T for the detection of brainstem infarctions.

**Methods:**

Data of patients with symptoms consistent with acute and subacute brainstem infarction and an MRI examination including standard axial DWI and thin-sliced sagittal DWI were retrospectively analyzed. Patients with the later diagnosis of a TIA, an inflammation or a tumor of the brainstem were excluded from analysis. Diffusion restrictions were identified by two independent raters blinded for the final clinical diagnosis in three separate reading steps: First, only axial DWI, secondly only sagittal DWI, and lastly both DWIs together. Presence and size of DWI-lesions were documented for each plane. Differences between the observers were settled in consensus in a separate joint reading.

**Results:**

Of 73 included patients, 46 patients were clinically diagnosed with brainstem infarction. Inter-observer agreement was excellent for the detection of brainstem lesions in axial and sagittal DWI (kappa = 0.94 and 0.97). In 28/46 patients (60.9%) lesions were detected in the axial plane alone, whereas in 6 more patients (73.9%) lesions were detected in the review of both sequences together. All lesions undetectable in the axial plane were smaller than 5 mm in cranio-caudal direction.

**Conclusions:**

Thin-sliced sagittal DWI in addition to axial DWI improves the detection rate of brainstem infarction with little additional expenditure of time.

## Introduction

Owing to its high sensitivity and specificity, diffusion weighted imaging (DWI) has substantially facilitated the diagnosis of brain infarction with single-shot echo planar imaging (EPI)-DWI as the current standard. Whereas the accuracy of DWI is excellent for larger infarcts, sensitivity is considerably lower in small infarcts. Especially brainstem infarctions have for long been associated with a higher rate of false-negative findings[1]. While some studies reported a high detection rate[2–6], other studies found high rates of false-negative DWI results for brainstem infarctions[1, 7–11].

Size of the lesions and susceptibility artifacts have been identified as confounding factors that attribute to false-negative scans[9].

Image quality is limited in the brainstem due to susceptibility artifacts caused by the proximity to the skull base and the mastoid. The signal of small infarcts may be blurred by the low spatial resolution of the conventional DWI. Still, in the brainstem, where efferent and afferent white matter fibers as well as nuclei of cranial nerves are so close to each other, these small infarcts may lead to grave symptoms. With the exception of some defined syndromes, diagnosis of brainstem infarctions is limited if based on clinical examination only[12].

To overcome this issue, acquisition of additional thin-sliced DWI sequences is recommended in some textbooks, but the usefulness of these recommendations has only been shown for an additional thin-sliced axial and coronal DWI[13, 14].

In our institution, for the suspicion of a brainstem infarction, an additional, thin-sliced EPI-DWI in the sagittal plane is routinely acquired.

We hypothesized that an additional thin-sliced sagittal DWI improves the detectability of brainstem infarctions compared to conventional axial DWI alone.

## Materials and methods

### Study population

This retrospective study was approved by the local ethics committee (Ethik-Kommission Ärztekammer Hamburg). Patient consent was not required by our local ethics committee for this de-identified database due to the retrospective nature of the study and the lack of patient interaction. Data of consecutive patients referred to our clinic between October 2008 and April 2012 that received a magnetic resonance imaging (MRI) examination for the initial clinical suspicion of a brainstem ischemia including axial DWI and sagittal DWI within 14 days after symptom onset were retrospectively analyzed. Exclusion criteria were symptoms resolving within 24 hours, therefore classifying the pathology as a transient ischemic attack, and non-ischemic pathologies as diagnosed over the clinical course that could cause any signal alteration of the brainstem like tumors or inflammation.

### Assessment of clinical data

Patients underwent intensive clinical examination including assessment of the stroke severity using the National Institute of Health Stroke Scale (NIHSS). The clinical diagnosis on discharge was employed as the gold standard to declare an infarction of the brainstem. Time symptom to DWI (t-STDWI) was calculated from onset of symptoms or the last time the patient was seen without symptoms and acquisition of the first DWI sequence.

### Imaging

Imaging was performed as part of the standard stroke protocol. DWI was performed using single-shot, multi-slice, spin-echo, EPI sequences in the axial and sagittal plane in the same scan on a 1.5 T MRI scanner (Siemens Magnetom Avanto, Erlangen, Germany). Single-shot EPI-DWI was obtained using diffusion gradients in three orthogonal directions, with b-values of 0, 500 and 1000 sec/mm^2^. Axial DWI covering the whole brain was performed using two different protocols: one with a TR/TE 3200/77 ms, matrix 128x96, thickness 5 mm, no gap, field of view 230 with an acquisition time of 56 seconds and another protocol with a TR/TE 4000/94 ms, matrix 128x128, slice thickness 5 mm, no gap, field of view 240 with an acquisition time of 44 seconds. Thinner sliced sagittal DWI only covered the width of the brainstem to decrease imaging time and was performed with a TR/TE 3500/80 ms, matrix 128x128, slice thickness 3 mm, no gap, field of view 230 with an acquisition time of 67 seconds.

### Image analysis

Two observers (M.H.S and R.R., both radiologists with each over 5 years of experience in stroke imaging) blinded to all patient information independently evaluated the DWI images and corresponding ADC maps in random order for the presence or absence of DWI-lesions on a work-station. Images were rated in three steps with a minimum of two weeks between each reading step: First, rating was performed on the axial images only (R_AX), secondly, on the sagittal images only (R_SAG), and lastly, the axial and sagittal images were rated together (R_ax+sag). Finally, in case of differing results between the observers, a consensus was reached by joint evaluation and these results were used for further analysis.

Maximal lesion diameters of consensus lesions in all image planes were measured manually on axial DWI and sagittal DWI. To determine lesion volumes, lesions were outlined on axial and sagittal DWI by manually adjusting the threshold of a seed growing algorithm. All image analyses were conducted using the software Analyze 11.0 (AnalyzeDirect, Inc., Overland Park, KS, USA).

Only lesion volumes derived from sagittal DWI scans entered further statistical analysis, since some lesions were not detectable on axial DWI.

For comparison with previous studies, time dependency of the detectability of infarctions was analyzed in a subgroup of patients with a t-STDWI ≤ 24h.

### Statistical analysis

Data is reported using standard descriptive statistics. Cohen’s kappa was calculated as a measure of interrater reliability. Spearman’s rank correlation test was used for correlations. Student’s t test and Mann-Whitney-U test were used as appropriate. All statistics were calculated using SPSS 19.0 (IBM SPSS Statistics for Windows, Armonk, NY: IBM Corp.). P-values < 0.05 were considered statistically significant.

## Results

A total of 95 patients fulfilled the inclusion criteria. Of these, a total of 22 patients were excluded. In detail, 18 patients were excluded due to resolving symptoms, implying a transient ischemic attack, 3 with inflammatory lesions and 1 with a tumor of the brainstem. Of the remaining 73 patients that entered analysis, 46 patients were clinically diagnosed with a brainstem infarction. The mean t-STDWI in the group with the clinical diagnosis of brainstem stroke was 56 h (range 1 h– 336 h).

The inter-observer reliability was excellent with kappa values of 0.94 (95% CI 0.86–1; p<0.001) for R_AX and 0.97 (95% CI 0.92–1; p<0.001) for R_SAG and R_ax+sag respectively.

One false positive lesion was found in R_AX by one observer but this particular lesion was declared to be negative in the consensus rating. No false positive lesion was detected by any rater in R_SAG or R_ax+sag.

DWI-positive lesions were detected in 28 (60.9%) of the 46 patients with brainstem infarctions in R_AX, in 33 (71.7%) patients in R_SAG and in 34 patients (73.9%) in R_ax+sag. Details on detection rates of lesions for all three reading steps are displayed in Table 1.

**Table 1:** Findings of the consensus rating of R_AX, R_SAG and R_ax+sag compared to the clinical diagnosis.

	R_AX		R_SAG		R_ax+sag		total
Clinical diagnosis	positive	negative	positive	negative	positive	negative	
**infarction**	28	18	33	13	34	12	46
**no infarction**	0	27	0	27	0	27	27
**total**	28	45	33	40	34	39	73

**Fig 1:**
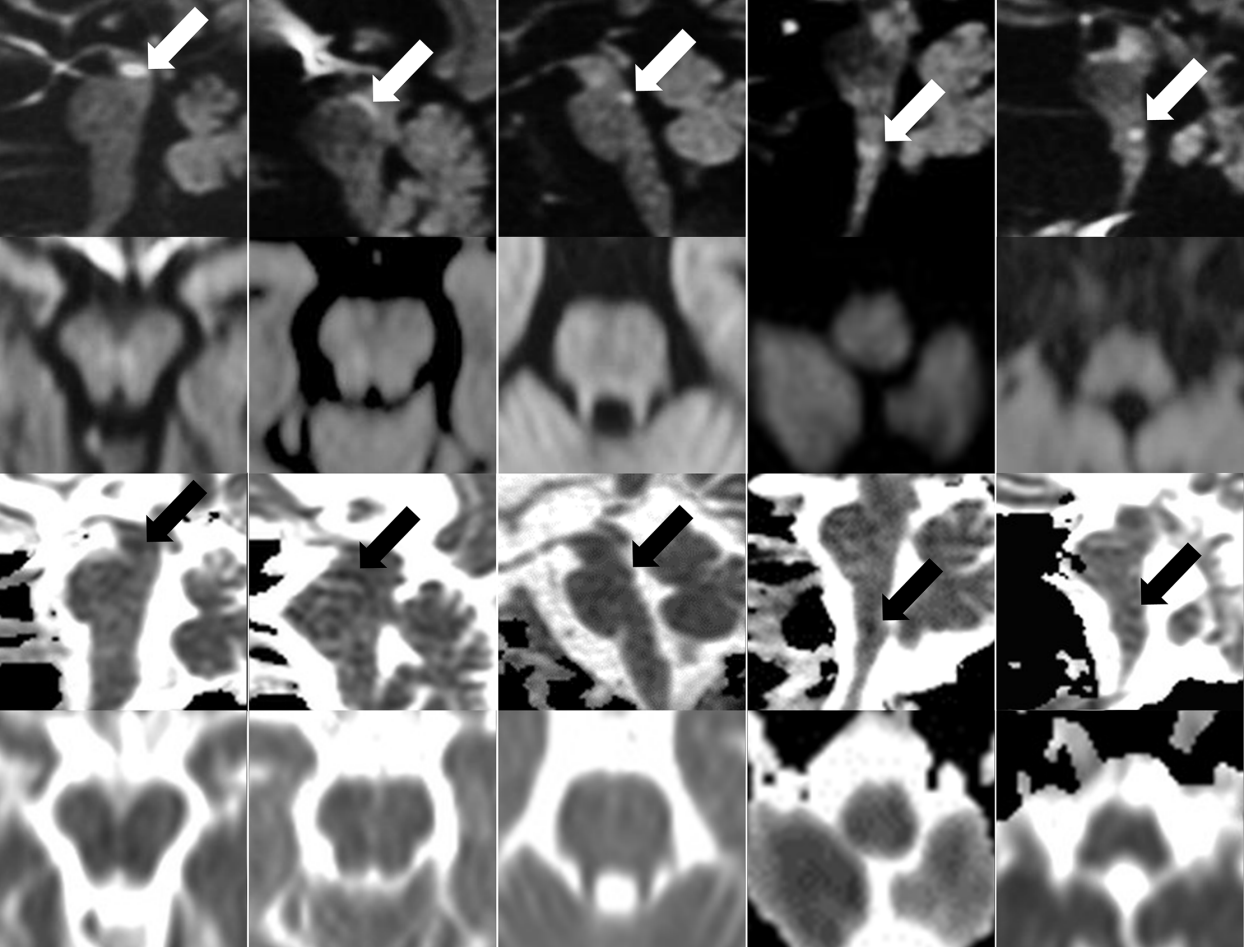
Lesions additionally detectable on sagittal DWI. Sagittal (first row) and corresponding axial (second row) B-1000 images and ADC-maps (third and fourth row) of the five DWI lesions (arrows) that were additionally detectable on the sagittal DWI (R_SAG).

**Fig 2:**
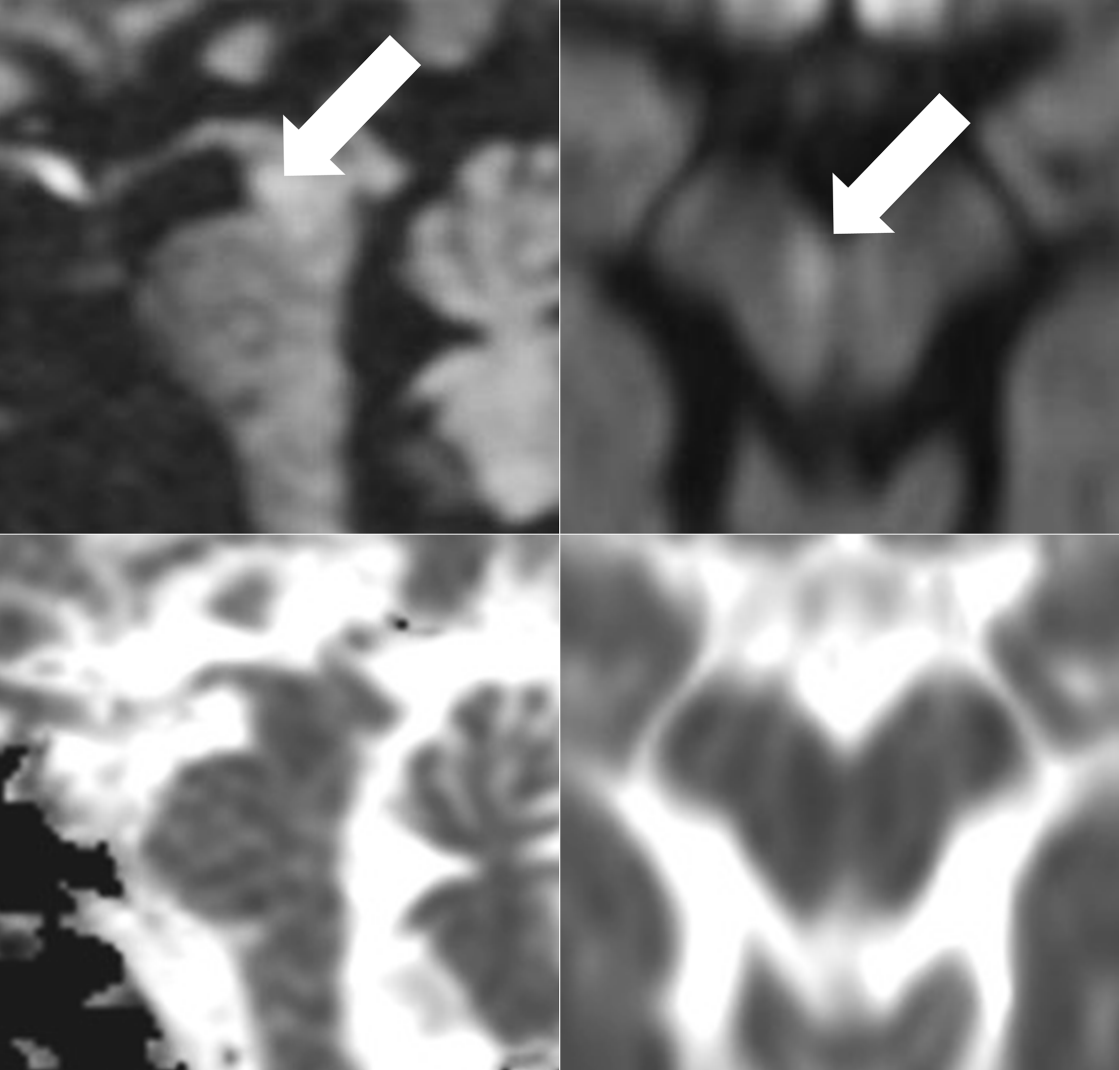
Lesion only detectable on review of both the axial and sagittal DWI together. B-1000 images and ADC-maps of the lesion (arrows) that was only detected on review both the axial (left) and the sagittal (right) DWI together (R_ax+sag).

All lesions detected in R_AX were also detected in R_SAG, whereas lesions in 5 more patients (15.2%) were detected in R_SAG with their respective location in the midbrain (n = 2), the pons (n = 1) and the medulla oblongata (n = 2) (Fig 1). One lesion in the midbrain was exclusively detected in R_ax+sag (Fig 2). The location of all lesions detected in R_ax+sag lesions was pontine in 14 patients, medullary in 8 patients, mixed pontine and mesencephalic in 6 patients, only mesencephalic in 5 patients and on the pontomedullary junction in 1 patient.

Sensitivity, Specificity, positive predictive value and negative predictive value for all three reading steps are presented in Table 2.

**Table 2:** Sensitivity, specificity, positive predictive value (PPV) and negative predictive value (NPV) for the detection of ischemic brainstem lesions in R_AX, R_SAG and R_ax+sag.

	Sensitivity	Specificity	PPV	NPV
**R_AX**	60.9 (45.4–74.5)	100.0 (84.5–100)	100.0 (85.0–100)	60.0 (44.4–73.9)
**R_SAG**	71.7 (56.3–83.5)	100.0 (84.5–100)	100.0 (87.0–100)	67.5 (50.8–80.9)
**R_ax+sag**	73.9 (58.6–85.2)	100.0 (84.5–100)	100.0 (87.4–100)	69.2 (52.3–82.5)

All values are percentages (95%confidence interval).

**Fig 3:**
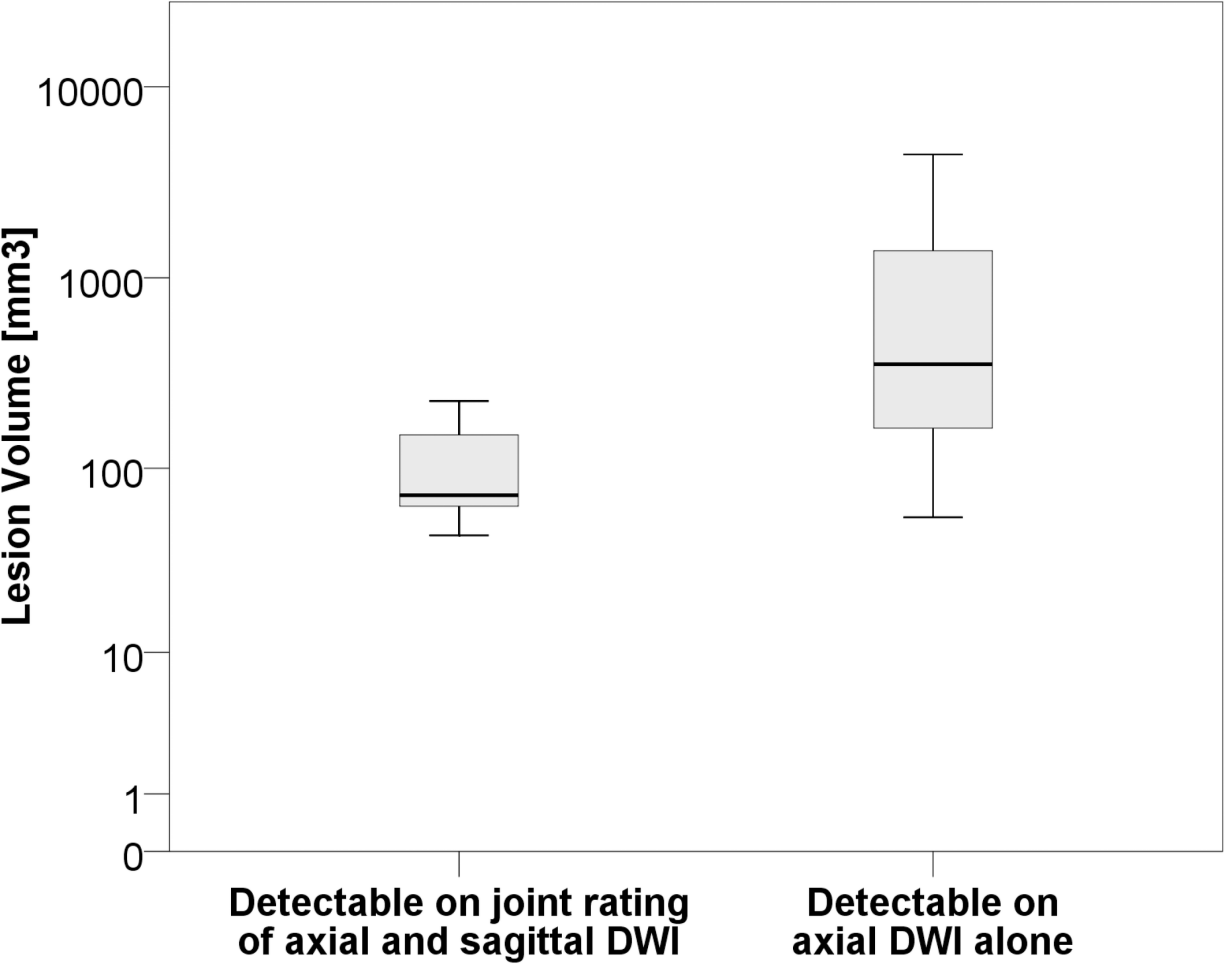
Lesion volumes of brainstem infarctions. Boxplots for the comparison of lesion volumes of brainstem infarctions detectable when reviewing the axial DWI only (R_AX) and when reviewing the axial DWI together with the additional sagittal DWI (R_ax+sag).

Volumes of lesions only detectable with the additional sagittal DWI were significantly smaller compared to those that could also be detected on the axial DWI alone (104.5 mm^3^ +/- 69.8 mm^3^ vs. 983.5 mm^3^ +/- 1237.8 mm^3^; p = 0.001) (Fig 3).

Comparing the diameters of the lesions, all lesions that were visible in R_SAG or R_ax+sag but undetected in R_AX were smaller than 5 mm in cranio-caudal direction. The cranio-caudal extend of the 28 lesions detected in R_AX was <5 mm in 13 cases and >5 mm in 15 cases. The cranio-caudal extension of the lesions detectable in R_AX was significantly larger than the ones only detectable in R_SAG and R_ax+sag (median diameter 6mm vs. 4mm; p = 0.006).

The t-STDWI was not significantly different in cases of positive compared to negative findings in R_ax+sag in all patients clinically diagnosed with a brainstem infarction (n = 46; mean t-STDWI 64.6 h +/- 80.8 h vs. 32.0 h +/-44.6 h, p = 0.19). This observation did not change in the subgroup analysis of patients with a t-STDWI ≤ 24h (n = 24; mean t-STDWI 7.8 h +/- 4.7 h vs. 9.0 h +/-8.9 h; p = 0.72).

The NIHSS in patients clinically diagnosed with a brainstem infarction was significantly higher in cases with a positive finding compared to those with a negative finding in R_ax+sag (NIHSS 3 vs. 1.5; p = 0.026). NIHSS did not differ between patients who exhibited positive findings in R_AX or R_SAG (Median NIHSS 3). The patient with the single lesion only detected in R_ax+sag presented with an NIHSS of 2.

NIHSS showed a significantly positive correlation with lesion volume with a Spearman’s rho of 0.55 (p = 0.001).

Details of each patient later clinically diagnosed with a brainstem infarction can be found in S1 Table.

## Discussion

Our data suggests that the acquisition of an additional thin-sliced sagittal DWI is equally specific as the axial DWI and improves the sensitivity to detect brainstem infarction with little expenditure of time.

While the use of an additional plane and thinner slices for brainstem infarctions is common and recommended even in textbooks[15, 16], evidence to back up the recommendations for thin sliced axial DWI is rare[13] and the benefit of an additional DWI acquired in the sagittal plane has only been anecdotal[17]. Recently it was shown that the combination of standard axial and thin-sectional coronal DWI facilitates the diagnosis of brainstem infarction but the effect was much smaller than in our study with only 3 out of 155 brainstem infarcts only detectable on the coronal DWI[14]. To the best of our knowledge this is the first study evaluating the recommendation for the acquisition in an additional sagittal plane systematically.

In our study, the detection rate of DWI lesions was comparable to that reported in previous studies. While some studies reported the detection rate to be even lower than within our study[10], other studies found higher detection rates, one of which reported the use of an additional sagittal DWI plane and, if needed, an additional coronal plane[6]. Sorimachi et al., who compared two DWI sequences, both acquired in axial plane with 6mm and 3mm slice thickness, reported a lower rate of false negatives of 16.2% for the detection of infratentorial strokes when using the 3mm DWI. However, they did not further discriminate between cerebellar and brainstem infarctions[13].

In our study, NIHSS showed a positive correlation with the DWI lesion volume. This is a common observation for supratentorial stroke, where correlations are usually even stronger than in our study[18] but can be found in the literature on brainstem infarctions as well[2], while other studies did not find such a correlation for infratentorial or posterior circulation stroke[3, 5]. This probably results from the fact that NIHSS favors symptoms from anterior circulation stroke over posterior circulation stroke[3].

In contrast to other studies that reported a reduction on the rate of false negative results with increasing t-STDWI[7, 19], we did not observe a time dependency of the detection rate in either the axial or the sagittal DWI.

The axial DWI used in our routine protocol had a lower spatial resolution than the sagittal DWI in the cranio-caudal direction. All lesions that were missed on the axial DWI were smaller than 5mm in the cranio-caudal direction and therefore might have been obscured by partial volume effects, i.e. the extent of these brainstem infarctions seemed to be too small to be detected with a slice thickness of 5mm.

Especially for infratentorial and spinal lesions, techniques to improve image quality have been proposed, for example multi-shot or readout segmented echo planar imaging[20, 21] and zoomed DWI[22]. A recent study found that a repeated conventional axial DWI with reversed phase-encoding polarity helped to discriminate infarctions from artifacts[23], a technique that is easily combined if not already inherent in the acquisition of the sagittal plane.

While performing MRI can be cost-effective in some instances of acute stroke imaging[24] it is not in most patients with TIA and minor stroke. An exception are patients with symptoms suggesting posterior circulation stroke[25]. Raising the sensitivity of the MRI from 60.9% to 73.9% while prolonging the scanning time by a minute only can increase the cost-effectiveness of the MRI in these patients further.

In the future, other thin sliced planes of DWI should be tested against each other, but so far, we suggest the use of the sagittal plane rather than the coronal or axial plane. In our opinion, DWI acquired in sagittal plane provides the best coverage of the whole length of the brainstem as well as the other posterior circulation territories, e.g. the cerebellum or the thalamus, with fewer slices. Particularly in these areas, even very small infarctions can lead to severe neurological impairment.

We explicitly recommend the combined acquisition of the sagittal and axial DWI, not substitution, since thin-sliced images acquired in the sagittal plane do not provide whole brain coverage and are therefore not sufficient to detect all supratentorial infarctions. In our experience, the additional DWI often provides the extra piece of information that is needed to decide on debatable findings and declare a subtle hyperintensity to be an artifact or a true infarction.

One limitation of our study is, that the patients included in our study presented with a wide range of t-STDWI, which might have influenced our results compared to other studies. Nevertheless, we did not find the DWI lesion detection rate to be dependent on the t-STDWI. Most other studies on brainstem or posterior circulation infarction excluded patients with a t-STDWI >24 h, which severely limits the implementation of their results into clinical practice[2–4, 6, 10, 13]. In most primary care centers, MRI scan time and availability are scarce resources. Patients that present with minor symptoms with the suspicion of brainstem infarcts might only receive an acute CT scan on admission to rule out hemorrhage and might subsequently receive an MRI scan in the following days. We therefore also included patients with a longer time span in our study to more closely resemble the clinical setting, which, in our opinion, strengthens the transferability of our observations into clinical practice.

Another limitation might be that the etiology of DWI lesions in the brain is diverse and includes vascular as well as non-vascular lesions[26]. Transient isolated brainstem symptoms frequently precede vertebrobasilar stroke[27] and in up to 29% of patients with a transient ischemic attack with clinical deficits linked to the brainstem DWI positive lesions can be seen[28]. Excluding these patients led to a relatively small number of patients that were analyzed and we probably altered the observed sensitivity, specificity, positive predictive value and negative predictive value by doing so. Nevertheless, as we sought to examine the value of an additional sagittal DWI in definite brainstem stroke we decided to exclude these patients to strengthen the validity of our observations, because these etiologies are easily distinguished over the clinical course. A study to examine the value of an additional sagittal DWI in patients suffering from TIA of the posterior circulation therefore is desirable.

Also, the use of clinical evaluation as a „gold standard” is suboptimal because the results of the DWI might have influenced the clinical diagnosis itself. Still, leaving the histopathologic examination aside, focused bedside examination is considered the most accurate modality to establish the diagnosis, especially for the differentiation between infratentorial stroke and more benign disorders in cases of minor symptoms[29]. Lacking other standards of reference this method has been applied in virtually every other study evaluating DWI in stroke.

Lastly, we only included images acquired at a field strength of 1.5T in our study, where image noise is higher compared to 3T. However, although the use of a higher field strength would reduce image noise, it would do so on the cost of stronger susceptibility artifacts which are especially problematic in infratentorial lesions[30].

In conclusion, this study shows that the number of brainstem infarctions that can be detected increases, when adding a thin-sliced sagittal DWI to the standard axial DWI. Given the overall low sensitivity of DWI for the detection of brainstem infarctions and the relatively short additional acquisition time of a sagittal DWI, it can easily and cost-effectively be implemented as an addition to the regular axial DWI in clinical routine examination protocols for suspected brainstem infarction.

## Supporting information

S1 Table Characteristics of 46 patients diagnosed with brainstem infarction with and without a DWI positive lesion. (DOCX)
