# Supplementary material for: Improved detectability of acute and subacute brainstem infarctions by combining standard axial and thin-sliced sagittal DWI
Source: PLoS One. 2018 Jul 3;13(7):e0200092. doi: 10.1371/journal.pone.0200092 (PMC6029789; doi:10.1371/journal.pone.0200092)
Supplement: S1 Table — (DOCX) [file pone.0200092.s001.docx]

| **S1 Table: Characteristics of 46 patients diagnosed with brainstem infarction with and without a DWI positive lesion.** | | | | | | |
| --- | --- | --- | --- | --- | --- | --- |
| **No.** | **Sex** | **Age [years]** | **NIHSS** | **t-STDWI [h]** | **visibility^a^** | **lesion location** |
| 1 | M | 72 | 2 | 136 | 1 | Left paramedian midbrain |
| 2 | M | 78 | 3 | 75 | 2 | left paramedian dorsal pons |
| 3 | F | 59 | 1 | 6 | 2 | left ventral medulla |
| 4 | M | 91 | 3 | 4 | 2 | right midbrain |
| 5 | F | 69 | 1 | 120 | 2 | left midbrain |
| 6 | F | 88 | 3 | 118 | 2 | left lateral medulla |
| 7 | M | 74 | 5 | 33 | 3 | left lateral medulla |
| 8 | M | 67 | 2 | 2 | 3 | right paramedian pons |
| 9 | M | 64 | 1 | 31 | 3 | right dorsal pons |
| 10 | F | 86 | 7 | 56 | 3 | right paramedian pons |
| 11 | M | 65 | 4 | 25 | 3 | central pontomedullary junction |
| 12 | M | 69 | 3 | 89 | 3 | left paramedian pons and right midbrain |
| 13 | F | 73 | 4 | 81 | 3 | left paramedian pons |
| 14 | M | 68 | 1 | 8 | 3 | left paramedian pons |
| 15 | F | 74 | 3 | 20 | 3 | right dorsal pons and right midbrain |
| 16 | M | 57 | 3 | 7 | 3 | left lateral medulla |
| 17 | M | 58 | 28 | 121 | 3 | bilateral pons and midbrain |
| 18 | M | 49 | 6 | 94 | 3 | bilateral dorsal pons and midbrain |
| 19 | F | 34 | 8 | 43 | 3 | left lateral medulla |
| 20 | M | 65 | 3 | 31 | 3 | right midbrain |
| 21 | F | 86 | 3 | 47 | 3 | right paramedian pons |
| 22 | F | 41 | 4 | 162 | 3 | bilateral pons and midbrain |
| 23 | M | 75 | 3 | 14 | 3 | right dorsal pons |
| 24 | F | 66 | 2 | 8 | 3 | left dorsal pons |
| 25 | F | 72 | 28 | 14 | 3 | bilateral pons and midbrain |
| 26 | F | 84 | 0 | 7 | 3 | left midbrain |
| 27 | F | 72 | 2 | 6 | 3 | left paramedian pons |
| 28 | M | 75 | 2 | 266 | 3 | left lateral medulla |
| 29 | F | 71 | 13 | 7 | 3 | left pons |
| 30 | M | 48 | 0 | 4 | 3 | right dorsal pons |
| 31 | M | 76 | 9 | 336 | 3 | bilateral pons |
| 32 | F | 63 | 0 | 151 | 3 | left lateral medulla |
| 33 | M | 64 | 3 | 6 | 3 | left lateral medulla |
| 34 | M | 53 | 8 | 4 | 3 | left dorsal pons |
| 35 | F | 70 | 2 | 1 | 0 |  |
| 36 | M | 63 | 9 | 1 | 0 |  |
| 37 | M | 76 | 1 | 2 | 0 |  |
| 38 | M | 66 | 0 | 7 | 0 |  |
| 39 | F | 68 | 3 | 9 | 0 |  |
| 40 | F | 56 | 1 | 70 | 0 |  |
| 41 | F | 55 | 3 | 24 | 0 |  |
| 42 | M | 60 | 2 | 24 | 0 |  |
| 43 | F | 75 | 0 | 6 | 0 |  |
| 44 | M | 49 | 0 | 97 | 0 |  |
| 45 | M | 46 | 3 | 7 | 0 |  |
| 46 | F | 72 | 2 | 200 | 0 |  |
| ^a^ 1: Diffusion restriction detected only on joint review of axial and sagittal DWI (detected in R_ax+sag but not R_SAG or R_AX); 2: Diffusion restriction detected on review of sagittal but not on axial DWI (detected on R_ax+sag and R_SAG but not R_AX); 3: Diffusion restriction visible on separate reviews of both axial and sagittal DWI (visible in all reading steps, R_AX, R_SAG and R_ax+sag); 0: No diffusion restriction detected. | | | | | | |
